# Association of Vascular Endothelial Growth Factor A +936 C/T Polymorphism With Non‐Alcoholic Fatty Liver Disease in a Bangladeshi Population: A Case‐Control Study

**DOI:** 10.1002/hsr2.73494

**Published:** 2026-11-05

**Authors:** Rakibul Hasan Shanto, Imran Hossain, Ahmad Niaz Rahman, Fazle Rabbi, Anik Dhar, Sheikh Faisal Asadullah Mahdi, Tarhima Jahan Jerin, Md. Samsul Arafin, Mamun Al Mahtab, Sheikh Zahir Raihan

**Affiliations:** ^1^ Department of Clinical Pharmacy and Pharmacology, Faculty of Pharmacy University of Dhaka Dhaka Bangladesh; ^2^ Department of Pharmacy, Faculty of Biological Science and Technology Jashore University of Science and Technology Jashore Bangladesh; ^3^ Department of Pharmacy University of Asia Pacific Dhaka Bangladesh; ^4^ Department of Biotechnology, Faculty of Agriculture Bangladesh Agricultural University Mymensingh Bangladesh; ^5^ Department of Hepatology Sher‐E‐Bangla Medical College Hospital Barishal Bangladesh; ^6^ Department of Hepatology Bangladesh Medical University Dhaka Bangladesh

**Keywords:** +936 C/T polymorphism, angiogenesis, non‐alcoholic fatty liver disease, rs3025039, single nucleotide polymorphism, vascular endothelial growth factor A

## Abstract

**Background and Aims:**

Non‐Alcoholic Fatty Liver Disease (NAFLD) ranges from hepatic steatosis to advanced cirrhosis. Polymorphisms in the vascular endothelial growth factor A (VEGFA) gene can change its expression, potentially altering angiogenesis and advancing disease stage. This study aimed to evaluate the association between the VEGFA +936 C/T polymorphism and susceptibility to NAFLD in a Bangladeshi population.

**Methods:**

A case‐control study was conducted including 100 NAFLD patients and 100 controls. NAFLD was diagnosed by ultrasonography. Genotyping was performed using allele‐specific polymerase chain reaction followed by gel electrophoresis. Biochemical parameters were measured using standard enzymatic methods following the manufacturer's instructions.

**Results:**

The frequency of the T allele was significantly higher in NAFLD patients compared to controls (22.5% vs. 12%). The control genotype distribution was consistent with Hardy‐Weinberg equilibrium (*χ* ^2 ^= 0.2812, *p *= 0.60). The Cochran–Armitage trend test indicated an association between increasing T‐allele dosage and NAFLD (*Z* = 2.68, *p *= 0.007). In logistic regression, the T‐allele was associated with NAFLD in the crude additive model (OR = 2.05, 95% CI: 1.20–3.51, *p *= 0.009) and after adjustment for age, sex, BMI, and fasting blood glucose (OR = 2.14, 95% CI: 1.13–4.08, *p *= 0.02). Under the dominant model, the association remained significant in both crude (OR = 2.27, 95% CI: 1.22–4.22, *p *= 0.01) and adjusted analyses (OR = 2.51, 95% CI: 1.18–5.33, *p *= 0.02). NAFLD patients showed significantly higher blood pressure, fasting blood glucose, HbA1c, total cholesterol, ALT, and AST, with lower HDL, while body mass index didn't differ significantly between groups.

**Conclusion:**

This study demonstrated a significant association between the VEGFA +936 C/T polymorphism and susceptibility to NAFLD in a Bangladeshi population. However, due to the small sample size and limited number of TT individuals, further large‐scale and functional studies are required to validate these findings.

## 1 Introduction

Non‐alcoholic fatty liver disease (NAFLD) affects approximately 25% of the adult population worldwide and represents a major contributor to global disease burden, with an estimated 48.31 million cases and 97,404 deaths among adults aged 20–49 years in 2021. It is a disorder that progresses from simple steatosis to non‐alcoholic steatohepatitis (NASH), fibrosis, and ultimately cirrhosis [1, 2, 3]. NAFLD can lead to multiple complications, including hepatocellular carcinoma, type 2 diabetes mellitus, chronic kidney disease, cardiovascular disease, metabolic syndrome, gallstone formation, and an increased risk of extrahepatic cancers [4, 5, 6, 7, 8, 9]. Although the prevalence of NAFLD varies among ethnic groups, it is highly prevalent in Asia, and South Asians are particularly susceptible to developing fatty liver even at a lower body mass index (BMI) [10, 11].

NAFLD develops when excess fat accumulates in liver cells due to impaired lipid metabolism. Chronic inflammation and oxidative stress further exacerbate the condition. Emerging evidence suggests that, beyond metabolic dysregulation, angiogenesis is a key driver of NAFLD progression. Vascular endothelial growth factor A (VEGFA) is a central mediator of angiogenesis, regulating endothelial cell proliferation and new blood vessel formation [12, 13]. Several studies have shown that inhibiting VEGF receptor 2 in obese mice improves multiple metabolic parameters, highlighting the important role of VEGF signaling in the progression of NAFLD [14, 15].

The VEGFA gene is located on chromosome 6p21.3. It belongs to the VEGF gene family, which consists of seven members: VEGFA, VEGFB, VEGFC, VEGFD, VEGFE, VEGFF, and placental growth factor [16, 17]. Among them, VEGFA promotes capillary permeability, cell migration, and endothelial cell proliferation, leading to the formation of new blood vessels [18, 19]. VEGFA can stimulate hepatic stellate cell activation, potentially worsening disease progression [20].

NAFLD development and progression are influenced by various factors, including genetic predisposition, environmental exposures, and metabolic disorders [21, 22, 23]. Several polymorphisms in the VEGFA gene can alter VEGFA expression, potentially exacerbating NAFLD [18, 24]. Among these genetic variations, single‐nucleotide polymorphisms (SNPs) are the most common and clinically significant. The +936 C/T SNP (rs3025039) in the 3′‐UTR of VEGFA affects both circulating plasma levels and tumor tissue expression of VEGFA [25, 26]. This region also plays a role in post‐transcriptional regulation. Moreover, this SNP has been associated with several diseases, including coronary artery disease, diabetic retinopathy, primary myelofibrosis, and liver‐related conditions such as biliary atresia [27, 28, 29, 30]. Furthermore, other VEGFA polymorphisms, such as −460 T/C (rs833061), have been linked to an increased risk of NAFLD. In the same study, the +936 C/T polymorphism was also evaluated, where individuals with the CT or TT genotype showed a higher prevalence of NAFLD [31]. However, this association has not been explored in the Bangladeshi population.

In 2023, a multi‐society Delphi consensus introduced metabolic dysfunction‐associated steatotic liver disease (MASLD) as the replacement term for NAFLD and proposed a new definition based on the presence of hepatic steatosis and at least one cardiometabolic risk factor [32]. In the present study, we retain the term NAFLD because participants were recruited and classified according to the study protocol using ultrasonographic evidence of hepatic steatosis together with exclusion of significant alcohol consumption and other specified liver diseases. Therefore, our study follows the previous NAFLD framework rather than the newer MASLD diagnostic criteria.

The prevalence of NAFLD is steadily increasing in Bangladesh. Therefore, identifying the underlying genetic factors may help in early disease diagnosis and enable better treatment strategies for the population. However, evidence regarding the association between the VEGFA +936 C/T polymorphism and NAFLD remains scarce, and to date, this association has not been investigated in the Bangladeshi population. Accordingly, the study aimed to determine whether the VEGFA +936 C/T (rs3025039) polymorphism is associated with susceptibility to NAFLD in the Bangladeshi population. Based on previous findings, we hypothesized that individuals carrying the polymorphic genotypes have a higher risk of developing NAFLD among Bangladeshi adults. This research provides baseline evidence for future studies and may help improve NAFLD prevention and treatment strategies, particularly in South Asian populations.

## 2 Materials and Methods

### 2.1 Patients

A case‐control study design was implemented. Peripheral blood samples were collected from May 2025 to December 2025 in EDTA‐containing tubes from 100 patients with NAFLD at BIRDEM General Hospital and 100 unrelated controls recruited from tertiary‐care hospitals in Dhaka city, including BIRDEM General Hospital, Dhaka Medical College Hospital, and Bangladesh Medical University, who were found not to have NAFLD. These recruitment sites were predefined in the study procedures. Participants were recruited on a non‐consecutive basis during the study period. During the recruitment period, the research team regularly visited the participating hospitals, screened individuals according to the predefined eligibility criteria, and enrolled eligible participants as cases or controls based on the presence or absence of NAFLD. Relevant clinical information and medical histories were recorded using a structured data collection form. Participants were included if they were adults aged ≥ 18 years with a diagnosis of NAFLD, established based on clinical evaluation, biochemical parameters, and imaging modalities. NAFLD diagnosis was confirmed by ultrasonography in all cases. Exclusion criteria included alcohol consumption exceeding 20 g/day, presence of viral hepatitis, drug‐induced liver injury, autoimmune liver disease, other recognized secondary causes of hepatic steatosis, severe comorbid conditions (including liver failure, heart failure, or renal failure), and pregnancy or lactation. A uniform alcohol consumption threshold of 20 g/day was applied to both sexes as a conservative and standardized criterion during participant recruitment. Alcohol consumption was assessed by self‐report during the recruitment interview. Controls were adults without ultrasonographic evidence of fatty liver or other hepatic comorbidities and without the exclusion conditions, and were recruited from the participating hospitals during the study period.

Anthropometric and biochemical measurements were performed using standard protocols. Diabetes, hypertension, and obesity were recorded as categorical variables based on the predefined study criteria. Hypertension was defined as systolic blood pressure ≥ 140 mmHg and/or diastolic blood pressure ≥ 90 mmHg. Obesity was defined as BMI ≥ 25 kg/m^2^ according to South Asian‐specific recommendations [33]. Diabetes status was determined from participants' clinical history and available medical records, including a history of physician‐diagnosed diabetes. Participants with previously diagnosed diabetes were classified as diabetic regardless of their current fasting glucose or HbA1c levels, including those receiving antidiabetic treatment. Anthropometric and biochemical measurements, along with relevant clinical information, were collected or recorded during the recruitment period. The distributions of diabetes, hypertension, and obesity are presented in Table 1.

**Table 1:** Demographic and clinical characteristics of participants.

Parameter	NAFLD cases (*n* = 100)	Controls (*n* = 100)	*p* value
Age (years)	39.56 ± 9.89	37.9 ± 5.08	0.14^a^
BMI (kg/m^2^)	26 (22.63–28)	25 (22.45–27)	0.48^b^
AST (IU/L)	30 (24–40.75)	29 (23–32)	0.02^b^
ALT (IU/L)	38 (26–57.75)	34 (30–38)	0.004^b^
AST/ALT ratio	0.83 (0.73–0.97)	0.82 (0.77–0.87)	0.50^b^
TC (mg/dL)	195 (168.3–221.5)	167.5 (160–175)	< 0.001^b^
TG (mg/dL)	193.5 (161–237.8)	140 (130–230)	0.11^b^
HDL cholesterol (mg/dL)	38 (34–42.75)	44 (42.25–46)	< 0.001^b^
LDL cholesterol (mg/dL)	118 (95–139.5)	112 (105–119)	0.29^b^
Systolic blood pressure (mmHg)	125 (116.3–130)	120 (110–120)	< 0.001^b^
Diastolic blood pressure (mmHg)	80 (70–85)	80 (70–80)	0.006^b^
FBG (mmol/L)	5.45 (5–6.7)	4.9 (4.5–5)	< 0.001^b^
HbA_1c_ (%)	5.25 (4.8–6.32)	4.7 (4.4–5.1)	< 0.001^b^
Gender *n* (%)			
Male	52	54	0.89^c^
Female	48	46	
Residence *n* (%)			
Urban	70	69	0.88^c^
Rural	30	31	
Type 2 diabetes *n* (%)			
Yes	33	23	0.12^c^
No	67	77	
Hypertension *n* (%)			
Yes	19	10	0.07^c^
No	81	90	
Obesity *n* (%)			
Yes	57	48	0.20^c^
No	43	52

*Note:* Data are expressed as mean ± SD or median (interquartile range, IQR), and *n* (%) for categorical variables.

^a^ Unpaired *t*‐test.

^b^ Mann–Whitney *U* test.

^c^ *χ* ^2^ test.

Ethical approval was obtained from the Faculty of Pharmacy, University of Dhaka (Ref. No. Fa. Ph. E/055/2025), and all procedures were conducted in accordance with the Declaration of Helsinki. The approved study procedures covered participant recruitment and sample collection at the three specified hospitals. Written informed consent was obtained from all participants prior to enrollment.

### 2.2 Genotyping

Genomic DNA was extracted from blood samples using the TIANamp Blood DNA Kit (TIANGEN). The concentration and purity of the extracted DNA were assessed using the EzDrop1000 micro‐volume spectrophotometer (Blue‐Ray Biotech, Taipei, Taiwan). Genomic DNA was stored at −20°C. The VEGFA (+936) polymorphism was identified by amplifying a 126 bp DNA fragment using an allele‐specific polymerase chain reaction (AS‐PCR). For each DNA sample, two separate PCR reactions were performed: one using the F‐Wild primer (5′‐ GGTCGGGTGACCCAGCAC‐3′) and the other using the F‐Mutant primer (5′‐ GGTCGGGTGACCCAGCAT‐3′), each paired with a common reverse primer (5′‐ GGGTGGGTGTGTCTACAGGA‐3′). A deliberate mismatch was present at the third nucleotide from the 5′ end of both allele‐specific forward primers, in addition to the allele‐specific nucleotide at the 3′ terminus corresponding to the VEGFA +936 C/T polymorphism. To ensure DNA quality and PCR performance, each reaction also included an internal positive control primer pair (Forward: 5′‐AAGGAAGAGGAGACTCTGCGC‐3′ and Reverse: 5′‐TATGTGGGTGGGTGTGTCTACAGG‐3′), which consistently generated a 198 bp control band in all samples (Figure 1). The PCR reaction was performed in a total volume of 10 μL, containing 50 ng of genomic DNA, 5 μL of EmeraldAmp GT PCR Master Mix (Takara Bio Inc., Kusatsu, Japan), 0.2 μL of allele‐specific forward primer, 0.2 μL of common reverse primer, 0.5 μL positive‐control forward primer, 0.5 μL positive‐control reverse primer, and 3.1 μL of nuclease‐free water. The amplification process began with an initial denaturation at 96°C for 1 min, followed by 35 cycles of 96°C for 10 s, 66°C for 25 s, and 72°C for 25 s. The reaction was completed with a final extension at 72°C for 7 min. The amplified products were loaded onto a 2% agarose gel stained with ethidium bromide and visualized under UV transillumination. For genotyping quality control, six randomly selected samples were independently amplified using a separate primer set targeting the SNP‐containing region and sequenced using Sanger sequencing by the dideoxy chain‐termination method, yielding 100% concordance with the corresponding AS‐PCR genotype calls (Supporting Information S1: Figure S1). Laboratory personnel were blinded to case/control status during genotyping and sequencing, and cases and controls were genotyped in the same PCR batches, with samples from both groups run on the same PCR plates and agarose gels. In addition, blinded duplicate genotyping was performed for 10 randomly selected samples, with 100% concordance between duplicate genotype calls. All samples yielded an unambiguous genotype call, corresponding to a genotyping call rate of 100%.

**Figure 1:**
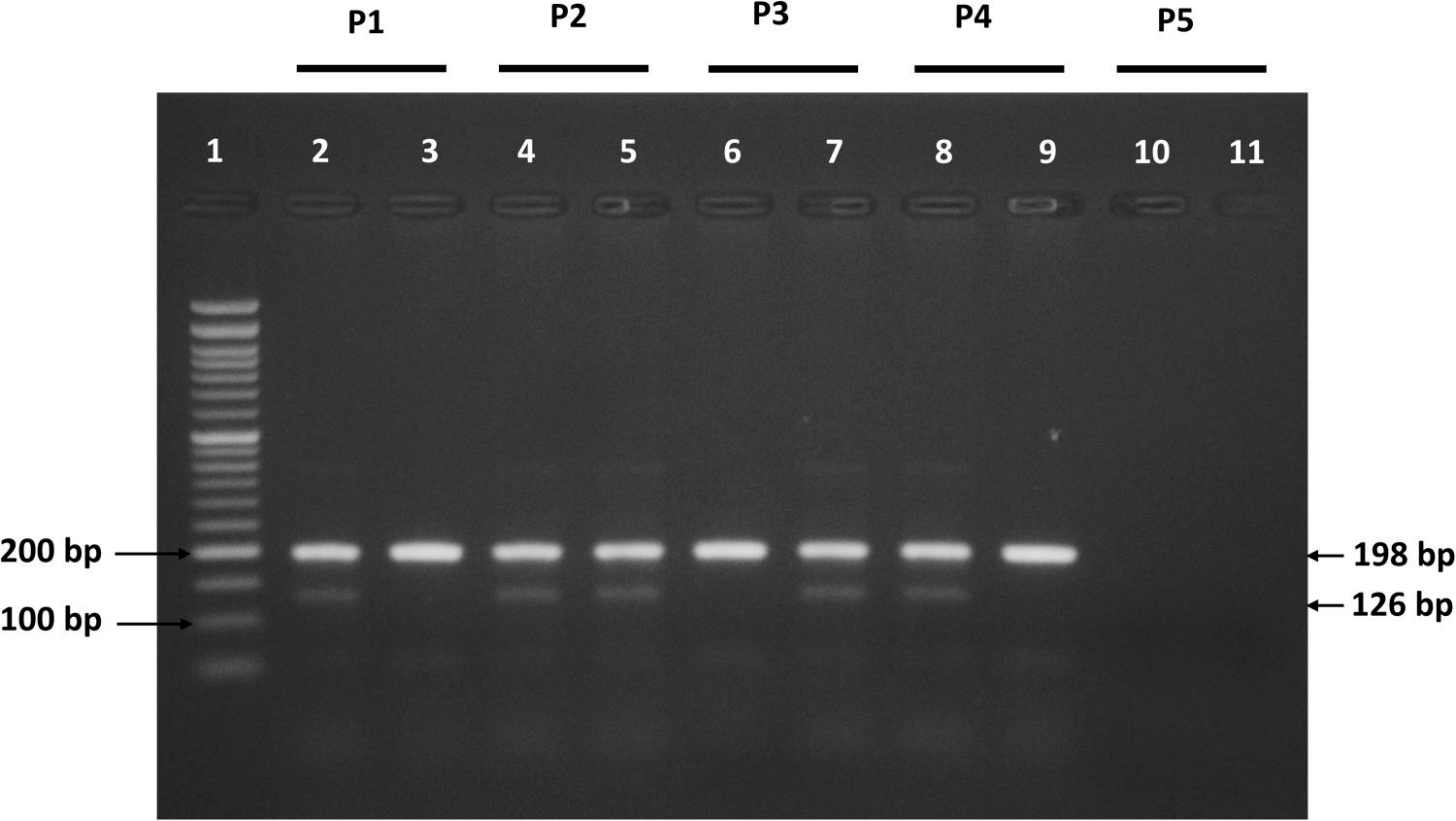
Allele‐specific PCR (AS‐PCR) analysis of the VEGFA +936 C/T polymorphism. A 126 bp DNA fragment indicates the presence of the corresponding allele, whereas the absence of amplification indicates the absence of that allele in the sample. A 198 bp band was used as an internal positive control to confirm DNA quality and successful amplification. Lane 1 contains a 50 bp DNA ladder, and lanes 2–11 represent individual samples. Samples were analyzed in pairs (P1–P5) using wild‐type and mutant‐specific forward primers, with the first lane of each pair representing the wild‐type (C)‐specific reaction and the second lane representing the mutant (T)‐specific reaction. Each PCR run included positive‐control forward and reverse primers, producing a 198‐bp band consistently across all samples. Among the sample pairs, P1 and P4 represent the homozygous CC genotype, P2 represents the heterozygous CT genotype, P3 represents the homozygous TT genotype, and P5 serves as the negative control. PCR products were separated on a 2% (w/v) agarose gel.

### 2.3 Statistical Analysis

Statistical analyses were performed using IBM SPSS Statistics version 26.0 (IBM Corp., Armonk, NY, USA) and GraphPad Prism version 10.3.0 (GraphPad Software, Boston, MA). The distribution of continuous variables was assessed using the Shapiro‐Wilk test. Continuous variables were summarized as mean ± standard deviation (SD) for normally distributed data and median (interquartile range [IQR]) for non‐normally distributed data, whereas categorical variables were summarized as frequencies and percentages. Continuous variables with a normal distribution were compared using the independent‐samples *t*‐test, whereas the Mann–Whitney U test was used for variables that were not normally distributed. Categorical variables were compared using the *χ* ^2^ test or Fisher's exact test, as appropriate. Genotype comparisons and their corresponding significance tests were performed using GraphPad Prism, which was also used for graphical visualization of the data. Genotype distributions among the control participants were assessed for Hardy–Weinberg equilibrium (HWE) using the *χ* ^2^ goodness‐of‐fit test with one degree of freedom. The Cochran–Armitage trend test was designated as the primary genetic association analysis during manuscript revision to assess the trend with increasing numbers of T alleles (0, 1, or 2) across the three VEGFA +936 C/T genotypes (CC, CT, and TT), corresponding to a log‐additive genetic model. The Cochran–Armitage trend test was performed using IBM SPSS Statistics. Additional allelic (T vs. C), heterozygous (CT vs. CC), homozygous (TT vs. CC), dominant (CT + TT vs. CC), and recessive (TT vs. CC + CT) genetic comparisons were performed as exploratory analyses. The C allele was considered the reference allele. Because these additional genetic models were not pre‐specified as primary comparisons, their *p* values were considered nominal. No formal adjustment for multiple comparisons was performed. A sensitivity analysis restricted to participants classified as non‐diabetic based on their clinical history and available medical records was performed using the dominant genetic model (CT + TT vs. CC).

Binary logistic regression was used to estimate crude and adjusted odds ratios (ORs) with 95% confidence intervals (CIs) for the additive and dominant genetic models. The additive model was coded as 0 = CC, 1 = CT, and 2 = TT, while the dominant model was coded as 0 = CC and 1 = CT/TT. Adjusted models included age, sex, BMI, and fasting blood glucose (FBG) as covariates. Logistic regression analyses were performed using IBM SPSS Statistics. All statistical tests were two‐sided, and an alpha level of 0.05 was used as the threshold for statistical significance. Effect estimates are reported with 95% CIs to convey the precision of the associations. Statistical reporting and interpretation were conducted in accordance with established recommendations for clinical research reporting [34]. This case‐control genetic association study was reported in accordance with the Strengthening the Reporting of Observational Studies in Epidemiology (STROBE) guidelines and the STROBE extension for Genetic Association studies (STREGA).

## 3 Results

Our study included 100 NAFLD patients and 100 controls, with no significant differences in age or gender distribution (*p* > 0.05). NAFLD patients had a higher BMI, although the difference was not statistically significant (*p *= 0.48). Systolic blood pressure (125 (116.3–130) vs. 120 (110–120), *p* < 0.001) and diastolic blood pressure (80 (70–85) vs. 80 (70–80), *p *= 0.006) were significantly higher in NAFLD patients compared to controls. Fasting blood glucose (5.45 (5–6.7) vs. 4.9 (4.5–5), *p* < 0.001), HbA1c (5.25 (4.8–6.32) vs. 4.7 (4.4–5.1), *p* < 0.001), total cholesterol (195 (168.3–221.5) vs. 167.5 (160–175), *p* < 0.001), and ALT (38 (26–57.75) vs. 34 (30–38), *p *= 0.004) were significantly elevated in NAFLD patients than controls. AST levels also differed significantly (30 (24–40.75) vs. 29 (23–32), *p *= 0.02), although the absolute difference was small. HDL cholesterol (38 (34–42.75) vs. 44 (42.25–46), *p* < 0.001) was significantly lower in NAFLD patients compared with controls. No significant differences were observed for triglycerides or LDL cholesterol between the two groups (*p* > 0.05).

Among the NAFLD cases, diabetes was present in 33% (33/100), hypertension in 19% (19/100), and obesity in 57% (57/100), compared with 23% (23/100), 10% (10/100), and 48% (48/100), respectively, among controls. None of these categorical metabolic characteristics differed significantly between the groups (diabetes, *p *= 0.12; hypertension, *p *= 0.07; obesity, *p *= 0.20) (Table 1).

Genotype distribution analysis revealed that the CC genotype was present in 61% of NAFLD patients and 78% of controls, whereas the CT genotype was more frequent in NAFLD patients (33% vs. 20%). The TT genotype was relatively rare in both groups (6% vs. 2%). The T‐allele frequency was 22.5% among NAFLD patients and 12.0% among controls. The T‐allele frequency among the controls was comparable to the reported South Asian minor allele frequency of 10.9% for rs3025039 based on NCBI dbSNP data [35]. The genotype distribution in the control group was consistent with HWE (*p *= 0.60) (Table 2).

**Table 2:** Distribution of VEGFA +936 C/T genotypes and alleles among NAFLD cases and controls.

Genotype frequency	NAFLD cases		Controls	
	Number	Frequency (%)	Number	Frequency (%)
CC	61	61	78	78
CT	33	33	20	20
TT	6	6	2	2
Allele frequency				
C	155	77.5	176	88
T	45	22.5	24	12

*Note:* Hardy–Weinberg equilibrium: *χ* ^2^ = 0.2812, *p* = 0.60 for control group.

The Cochran–Armitage trend test demonstrated a significant linear trend in the distribution of VEGFA +936 C/T genotypes between NAFLD patients and controls (*Z* = 2.68, *p *= 0.007), supporting a log‐additive association between the number of T alleles and NAFLD susceptibility.

Additional genetic‐model analyses were performed as exploratory comparisons. The T allele was more frequent among NAFLD patients than controls (22.5% vs. 12.0%) and showed a nominally significant association with NAFLD (OR = 2.13, 95% CI: 1.24–3.62, *p *= 0.006). Under the heterozygous model, the CT genotype was associated with higher odds of NAFLD compared with CC (OR = 2.11, 95% CI: 1.12–3.98, *p *= 0.02). Under the dominant model, individuals carrying at least one T allele (CT + TT) had higher odds of NAFLD compared with CC (OR = 2.27, 95% CI: 1.21–4.29, *p *= 0.009). The homozygous (TT vs. CC) and recessive (TT vs. CC + CT) comparisons were not statistically significant (Table 3). The homozygous TT genotype showed higher odds of NAFLD compared with the CC genotype (OR 3.84, 95% CI 0.92–19.06), although the association was not statistically significant (*p *= 0.14). Similarly, the recessive model (TT vs. CC + CT) did not show a significant association (OR 3.13, 95% CI 0.75–15.48, *p *= 0.28). Both TT‐related estimates should be interpreted cautiously because only two control participants had the TT genotype, resulting in wide confidence intervals.

**Table 3:** VEGFA +936 C/T polymorphism distribution under different inheritance models.

Model name	OR	95% CI	Test statistic (*Z*)	*p* value
Cochran‐Armitage trend test (primary analysis)	—	—	2.68	0.007
Allele (T vs. C)	2.129	1.24–3.62	—	0.006
Homozygous (TT vs. CC)	3.836	0.92–19.06	—	0.14
Heterozygous (CT vs. CC)	2.110	1.12–3.98	—	0.02
Dominant (CT + TT vs. CC)	2.267	1.21–4.29	—	0.009
Recessive (TT vs. CC + CT)	3.128	0.75–15.48	—	0.28

*Note:* Data are presented as odds ratios (ORs) with 95% confidence intervals (CIs). The Cochran–Armitage trend test was designated as the primary genetic association analysis during manuscript revision. The remaining genetic‐model comparisons were exploratory, and their *p* values are nominal. The *χ* ^2^ test was used for analyses with adequate cell counts, while Fisher's exact test was applied when expected counts were < 5. Comparisons involving the TT genotype should be interpreted cautiously because of the small number of TT individuals, particularly among controls (*n* = 2), resulting in wide confidence intervals and potentially unstable estimates.

In a sensitivity analysis restricted to participants classified as non‐diabetic based on their clinical history and available medical records, the dominant genetic model (CT + TT vs. CC) showed a significant association with NAFLD. The analysis included 67 cases and 77 controls. T‐allele carriers had higher odds of NAFLD than individuals with the CC genotype (OR = 2.50, 95% CI: 1.23–5.03, Fisher's exact test, *p *= 0.013).

In logistic regression analysis, the association remained significant after adjustment for age, sex, BMI, and fasting blood glucose (FBG). The adjusted additive model showed increased odds of NAFLD per additional T allele (OR = 2.14, 95% CI: 1.13–4.08, *p *= 0.02), while the adjusted dominant model showed a similar association for CT/TT versus CC (OR = 2.51, 95% CI: 1.18–5.33, *p *= 0.02) (Table 4).

**Table 4:** Crude and adjusted logistic regression analyses of the association between VEGFA +936 C/T polymorphism and non‐alcoholic fatty liver disease (NAFLD) under additive and dominant genetic models.

Model	Comparison/coding	Crude OR (95% CI)	Crude *p*	Adjusted OR (95% CI)	Adjusted *p*
Additive	0, 1, 2 T alleles	2.05 (1.20–3.51)	0.009	2.14 (1.13–4.08)	0.02
Dominant	CT + TT vs. CC	2.27 (1.22–4.22)	0.01	2.51 (1.18–5.33)	0.02

*Note:* Adjusted ORs were estimated using binary logistic regression adjusted for age, sex, BMI, and fasting blood glucose (FBG). For the additive model, genotype was coded as 0 = CC, 1 = CT, and 2 = TT. For the dominant model, genotype was coded as 0 = CC and 1 = CT/TT.

## 4 Discussion

In this case‐control study of Bangladeshi adults, we found that the VEGFA +936 C/T polymorphism was significantly associated with susceptibility to NAFLD. The Cochran–Armitage trend test demonstrated a significant linear trend in NAFLD susceptibility across the CC, CT, and TT genotypes. The association remained significant in logistic regression analyses under both additive and dominant models after adjustment for age, sex, BMI, and fasting blood glucose (FBG), supporting an association between the VEGFA +936 T allele and NAFLD susceptibility.

In this study, the study groups were comparable in age and homogeneous in terms of gender. NAFLD patients exhibited significantly higher blood pressure compared to controls, whereas BMI was higher in cases but did not reach statistical significance. Similar results have been reported in other studies [36, 37, 38]. Moreover, NAFLD patients showed elevated fasting blood glucose and HbA1c, which is consistent with a previous study in a Chinese population that reported similar results [39]. NAFLD patients also showed significantly higher total cholesterol and lower HDL, while triglycerides and LDL were higher but not statistically significant, consistent with findings from studies in Bangladeshi and Nepalese populations [36, 37]. Elevated levels of ALT and AST were also observed in NAFLD patients, consistent with previous studies [40, 41].

We found that the CC genotype was the most prevalent, while the TT genotype was the least common in both groups. The frequency of the T allele was significantly higher in NAFLD patients compared to controls (22.5% vs. 12%). The Cochran–Armitage trend test demonstrated a significant linear trend in genotype distribution between the two groups (*Z* = 2.68, *p *= 0.007), supporting a log‐additive association between the number of T alleles and NAFLD susceptibility.

Additional genetic‐model analyses showed that the T allele was associated with higher odds of NAFLD in the allelic analysis (OR = 2.13, 95% CI: 1.24–3.62, *p *= 0.006). The heterozygous CT genotype was also associated with higher odds compared with CC (OR = 2.11, 95% CI: 1.12–3.98, *p *= 0.02), while the dominant model showed a similar association (OR = 2.27, 95% CI: 1.21–4.29, *p *= 0.009). These findings should be interpreted as secondary genetic‐model analyses rather than independent primary evidence.

The biological mechanism underlying the association between the VEGFA +936 C/T polymorphism and NAFLD remains uncertain. Renner et al. [26] reported lower circulating VEGFA plasma levels among 936T allele carriers, suggesting that this variant may influence VEGFA availability. Altered VEGFA signaling could potentially affect angiogenesis and vascular responses involved in liver disease; however, this possibility remains speculative because VEGFA levels were not measured in the present study. Further functional studies are needed to clarify whether and how rs3025039 influences VEGFA‐related pathways in NAFLD.

For the homozygous polymorphic genotype (TT), the estimated odds ratio was 3.84 (95% CI: 0.92–19.06; *p*= 0.14), with the wide confidence interval reflecting the small number of TT individuals. Similarly, no statistically significant association was observed under the recessive model (TT vs. CC + CT; OR = 3.13, 95% CI: 0.75–15.48; *p *= 0.28).

To our knowledge, this is the first study to investigate the association between the VEGFA +936 C/T polymorphism and NAFLD in a Bangladeshi population. A similar association was previously reported in a case‐control study conducted in a Hubei Han Chinese population by Wu et al. [31], which included 246 NAFLD patients and 341 controls. In that study, the VEGFA +936 C/T (rs3025039) polymorphism was significantly associated with NAFLD risk. Compared with the CC genotype, individuals with the CT genotype had increased odds of NAFLD (adjusted OR = 1.64, 95% CI: 1.24–2.34, *p *= 0.009), while those with the TT genotype also showed increased odds (adjusted OR = 2.29, 95% CI: 1.21–4.57, *p *= 0.014). In the dominant model, carriers of the T allele (CT/TT) had significantly higher odds of NAFLD than individuals with the CC genotype (adjusted OR = 1.89, 95% CI: 1.41–2.82, *p* < 0.001).

Our findings are broadly consistent with this previous observation. In the present Bangladeshi population, the T allele was more frequent among NAFLD patients than controls (22.5% vs. 12.0%), and the Cochran–Armitage trend test demonstrated a significant linear trend across the three genotypes (*p *= 0.007). The adjusted additive model showed that each additional T allele was associated with higher odds of NAFLD (OR = 2.14, 95% CI: 1.13–4.08, *p *= 0.02). Similarly, the adjusted dominant model showed increased odds among T‐allele carriers (CT/TT) compared with CC homozygotes (OR = 2.51, 95% CI: 1.18–5.33, *p *= 0.02). Thus, the direction of association observed in our study is consistent with the findings of Wu et al. [31], despite differences in the study populations and analytical approaches.

The consistency in the direction of association across these two populations provides preliminary support for a potential relationship between the VEGFA +936 C/T polymorphism and NAFLD susceptibility. However, the available evidence remains limited, and the present findings should be interpreted cautiously given the relatively small sample size and the low frequency of the TT genotype, particularly among controls.

Overall, our findings demonstrate a significant association between the VEGFA +936 C/T polymorphism and susceptibility to NAFLD in this population. The significant Cochran–Armitage trend test supports a linear relationship between the number of T alleles and NAFLD susceptibility, while the association remained significant under adjusted additive and dominant logistic regression models. These findings provide preliminary evidence of a genetic association between VEGFA +936 C/T variation and NAFLD susceptibility. Larger, multi‐center studies with functional evaluation are required to validate these findings and clarify the underlying biological mechanisms.

Several limitations should be considered when interpreting our findings. First, a power calculation was not performed before the study initiation, and the sample size was relatively small. Given the 12% minor allele frequency observed among controls, the study had limited power to detect modest genetic effects, such as an odds ratio of 1.5. Therefore, the observed association may overestimate the underlying effect size, and a smaller true effect cannot be excluded. Second, the case group blood samples were collected at BIRDEM General Hospital, and control samples were collected from tertiary‐level hospitals in Dhaka city. Diabetes mellitus was present in 33% of cases and 23% of controls, although the difference was not statistically significant (*p *= 0.12). Therefore, differences in referral patterns and underlying metabolic characteristics between cases and controls cannot be excluded and may have introduced some degree of selection bias. Third, this study did not determine plasma VEGFA levels, preventing the assessment of the functional effect of this polymorphism. Finally, NAFLD diagnosis was based on ultrasonography without assessment of steatosis grade or histological severity, and fibrosis status was not evaluated. Consequently, the study could not assess associations between the VEGFA +936 C/T polymorphism and NAFLD severity or fibrosis stage. In addition, platelet counts were not collected, precluding calculation of fibrosis‐related indices such as FIB‐4.

## 5 Conclusion

This study demonstrated a significant association between the VEGFA +936 C/T polymorphism and susceptibility to NAFLD in a Bangladeshi population. The significant Cochran‐Armitage trend test supports an association between the increasing number of T alleles and NAFLD susceptibility, while the association remained significant under adjusted additive and dominant logistic regression models. These findings provide preliminary evidence of a genetic association between VEGFA +936 C/T variation and NAFLD susceptibility. However, due to the relatively small sample size and limited number of TT individuals, the results should be interpreted with caution and do not establish a causal relationship. Further large‐scale, multicenter, and functional studies are required to confirm these findings and clarify the underlying mechanisms and potential clinical relevance.

## Author Contributions

**Rakibul Hasan Shanto:** conceptualization, investigation, writing – original draft, methodology, formal analysis, software, data curation, visualization, validation, writing – review and editing. **Imran Hossain:** software, writing – review and editing, validation, data curation, visualization, conceptualization, methodology, writing – original draft. **Ahmad Niaz Rahman:** formal analysis, investigation. **Fazle Rabbi:** investigation, writing – review and editing. **Anik Dhar:** data curation, validation. **Sheikh Faisal Asadullah Mahdi:** visualization, data curation. **Tarhima Jahan Jerin:** formal analysis, data curation. **Md. Samsul Arafin:** validation, project administration. **Mamun Al Mahtab:** validation, project administration. **Sheikh Zahir Raihan:** conceptualization, writing – review and editing, visualization, validation, software, supervision, project administration, data curation, resources, formal analysis.

## Funding

The authors have nothing to report.

## Conflicts of Interest

The authors declare no conflicts of interest.

## Transparency Statement

Sheikh Zahir Raihan, the corresponding author, confirms that this manuscript provides an honest, accurate, and complete report of the study. No important parts of the study have been left out, and any differences from the planned study have been explained.

## Supporting information


**Figure S1:** Representative sequencing chromatograms showing CC, CT, and TT genotypes.

## Data Availability

The data supporting the findings of this study can be obtained from the corresponding author upon reasonable request.

## References

[1] N. C. Leite , C. A. Villela‐Nogueira , V. L. N. Pannain , et al., “Histopathological Stages of Nonalcoholic Fatty Liver Disease in Type 2 Diabetes: Prevalences and Correlated Factors: Histopathology of NAFLD in Diabetes,” Liver International 31, no. 5 (May 2011): 700–706, 10.1111/j.1478-3231.2011.02482.x. 21457442

[2] Z. Zhong , M. Xu , X. Luo , and J. Tan , “Global, Regional, and National Burden of Nafld in Adults Aged 20–49 Years (1990–2021): A Comprehensive Analysis of Trends, Decomposition, and Health Inequality,” BMC Gastroenterology 26, no. 1 (December 2025): 23, 10.1186/s12876-025-04497-4. PMC12801882 41350617

[3] S. Ciardullo , G. Grassi , G. Mancia , and G. Perseghin , “Nonalcoholic Fatty Liver Disease and Risk of Incident Hypertension: A Systematic Review and Meta‐Analysis,” European Journal of Gastroenterology & Hepatology 34, no. 4 (April 2022): 365–371, 10.1097/MEG.0000000000002299. PMC8876398 34678858

[4] K. Björkström , L. Widman , and H. Hagström , “Risk of Hepatic and Extrahepatic Cancer in NAFLD: A Population‐Based Cohort Study,” Liver International 42, no. 4 (April 2022): 820–828, 10.1111/liv.15195. PMC9306866 35152526

[5] E. Bonora and G. Targher , “Increased Risk of Cardiovascular Disease and Chronic Kidney Disease in NAFLD,” Nature Reviews Gastroenterology & Hepatology 9, no. 7 (July 2012): 372–381, 10.1038/nrgastro.2012.79. 22565095

[6] G. Forlani , C. Giorda , R. Manti , et al., “The Burden of NAFLD and Its Characteristics in a Nationwide Population With Type 2 Diabetes,” Journal of Diabetes Research 2016 (2016): 1–9, 10.1155/2016/2931985. PMC4829721 27123461

[7] P. Loria , A. Lonardo , S. Lombardini , et al., “Gallstone Disease in Non‐Alcoholic Fatty Liver: Prevalence and Associated Factors,” Journal of Gastroenterology and Hepatology 20, no. 8 (August 2005): 1176–1184, 10.1111/j.1440-1746.2005.03924.x. 16048564

[8] P. Wainwright and C. Byrne , “Bidirectional Relationships and Disconnects Between NAFLD and Features of the Metabolic Syndrome,” International Journal of Molecular Sciences 17, no. 3 (March 2016): 367, 10.3390/ijms17030367. PMC4813227 26978356

[9] N. Ratnasari , S. Nurdjanah , A. H. Sadewa , M. Hakimi , and Y. Yano , “Difference of Polymorphism VEGF‐Gene rs699947 in Indonesian Chronic Liver Disease Population,” PLoS One 12, no. 8 (August 2017): e0183503, 10.1371/journal.pone.0183503. PMC5570295 28837651

[10] K. Riazi , M. G. Swain , S. E. Congly , G. G. Kaplan , and A. A. Shaheen , “Race and Ethnicity in Non‐Alcoholic Fatty Liver Disease (NAFLD): A Narrative Review,” Nutrients 14, no. 21 (October 2022): 4556, 10.3390/nu14214556. PMC9658200 36364818

[11] S. Singh , G. N. Kuftinec , and S. Sarkar , “Non‐Alcoholic Fatty Liver Disease in South Asians: A Review of the Literature,” Journal of Clinical and Translational Hepatology 5, no. 1 (February 2017): 76, 10.14218/JCTH.2016.00045. PMC5411360 28507930

[12] M. Masarone , V. Rosato , M. Dallio , et al., “Role of Oxidative Stress in Pathophysiology of Nonalcoholic Fatty Liver Disease,” Oxidative Medicine and Cellular Longevity 2018, no. 1 (January 2018): 9547613, 10.1155/2018/9547613. PMC6016172 29991976

[13] S. Coulon , S. Francque , I. Colle , et al., “Evaluation of Inflammatory and Angiogenic Factors in Patients With Non‐Alcoholic Fatty Liver Disease,” Cytokine 59, no. 2 (August 2012): 442–449, 10.1016/j.cyto.2012.05.001. 22658783

[14] K. Sun , I. W. Asterholm , C. M. Kusminski , et al., “Dichotomous Effects of VEGF‐A on Adipose Tissue Dysfunction,” Proceedings of the National Academy of Sciences 109, no. 15 (April 2012): 5874–5879, 10.1073/pnas.1200447109. PMC3326476 22451920

[15] I. Elias , S. Franckhauser , and F. Bosch , “New Insights into Adipose Tissue VEGF‐A Actions in the Control of Obesity and Insulin Resistance,” Adipocyte 2, no. 2 (April 2013): 109–112, 10.4161/adip.22880. PMC3661112 23805408

[16] L. E. Harry and E. M. Paleolog , “From the Cradle to the Clinic: VEGF in Developmental, Physiological, and Pathological Angiogenesis,” Birth Defects Research Part C: Embryo Today: Reviews 69, no. 4 (December 2003): 363–374, 10.1002/bdrc.10024. 14745976

[17] H. Şamlı , B. Ç. Demir , A. Özgöz , M. A. Atalay , and G. Uncu , “Vascular Endothelial Growth Factor Gene 1154 G/A, 2578 C/A, 460 C/T, 936 C/T Polymorphisms and Association With Recurrent Pregnancy Losses,” Genetics and Molecular Research 11, no. 4 (2012): 4739–4745, https://pubmed.ncbi.nlm.nih.gov/23315815/. 10.4238/2012.December.17.6 23315815

[18] F. Liu , L. Luo , Y. Wei , et al., “Association of VEGFA Polymorphisms With Susceptibility and Clinical Outcome of Hepatocellular Carcinoma in a Chinese Han Population,” Oncotarget 8, no. 10 (March 2017): 16488–16497, 10.18632/oncotarget.14870. PMC5369979 28147320

[19] M. Scartozzi , L. Faloppi , G. Svegliati Baroni , et al., “VEGF and VEGFR Genotyping in the Prediction of Clinical Outcome for Hcc Patients Receiving Sorafenib: The ALICE‐1 Study,” International Journal of Cancer 135, no. 5 (September 2014): 1247–1256, 10.1002/ijc.28772. 24510746

[20] H. Shen , H. Yu , Q. Li , et al., “Hepatocyte‐Derived VEGFA Accelerates the Progression of Non‐Alcoholic Fatty Liver Disease to Hepatocellular Carcinoma via Activating Hepatic Stellate Cells,” Acta Pharmacologica Sinica 43, no. 11 (November 2022): 2917–2928, 10.1038/s41401-022-00907-5. PMC9622738 35508720

[21] L. A. Adams , S. W. White , J. A. Marsh , et al., “Association Between Liver‐Specific Gene Polymorphisms and Their Expression Levels With Nonalcoholic Fatty Liver Disease,” Hepatology 57, no. 2 (February 2013): 590–600, 10.1002/hep.26184. 23213074

[22] S. Rajak , S. Raza , A. Tewari , and R. A. Sinha , “Environmental Toxicants and NAFLD: A Neglected yet Significant Relationship,” Digestive Diseases and Sciences 67, no. 8 (August 2022): 3497–3507, 10.1007/s10620-021-07203-y. 34383198

[23] P. Golabi , J. M. Paik , T. Arshad , Y. Younossi , A. Mishra , and Z. M. Younossi , “Mortality of NAFLD According to the Body Composition and Presence of Metabolic Abnormalities,” Hepatology Communications 4, no. 8 (August 2020): 1136–1148, 10.1002/hep4.1534. PMC7395070 32766474

[24] Z. Gong , N. Qiao , Y. Gu , et al., “Polymorphisms of VEGFA Gene and Susceptibility to Hemorrhage Risk of Brain Arteriovenous Malformations in a Chinese Population,” Acta Pharmacologica Sinica 32, no. 8 (August 2011): 1071–1077, 10.1038/aps.2011.76. PMC4002532 21706043

[25] C. J. Watson , N. J. A. Webb , M. J. Bottomley , and P. E. C. Brenchley , “Identification of Polymorphisms Within the Vascular Endothelial Growth Factor (VEGF) Gene: Correlation With Variation in VEGF Protein Production,” Cytokine 12, no. 8 (August 2000): 1232–1235, 10.1006/cyto.2000.0692. 10930302

[26] W. Renner , S. Kotschan , C. Hoffmann , B. Obermayer‐Pietsch , and E. Pilger , “A Common 936 C/T Mutation in the Gene for Vascular Endothelial Growth Factor Is Associated With Vascular Endothelial Growth Factor Plasma Levels,” Journal of Vascular Research 37, no. 6 (2000): 443–448, 10.1159/000054076. 11146397

[27] F. Liu , J. Zeng , D. Zhu , et al., “Association of Polymorphism in the VEGFA Gene 3′‐ UTR +936T/C With Susceptibility to Biliary Atresia in a Southern Chinese Han Population,” Journal of Clinical Laboratory Analysis 32, no. 4 (May 2018): e22342, 10.1002/jcla.22342. PMC6817064 29251369

[28] W. Q. Ma , Y. Wang , X. Q. Han , Y. Zhu , and N. F. Liu , “Association of Genetic Polymorphisms in Vascular Endothelial Growth Factor With Susceptibility to Coronary Artery Disease: A Meta–Analysis,” BMC Medical Genetics 19, no. 1 (December 2018): 108, 10.1186/s12881-018-0628-3. PMC6031176 29973139

[29] L. Villani , A. Carolei , V. Rosti , et al., “Clinical Relevance of VEGFA (rs3025039) +936 C≫T Polymorphism in Primary Myelofibrosis: Susceptibility, Clinical Co‐Variates, and Outcomes,” Genes 12, no. 8 (August 2021): 1271, 10.3390/genes12081271. PMC8393853 34440447

[30] L. Han , L. Zhang , W. Xing , et al., “The Associations Between VEGF Gene Polymorphisms and Diabetic Retinopathy Susceptibility: A Meta‐Analysis of 11 Case‐Control Studies,” Journal of Diabetes Research 2014 (2014): 1–10, 10.1155/2014/805801. PMC4020464 24868559

[31] P. Wu , Y. Hua , S. Tan , M. Li , S. Yongxiang , and G. Fang , “Interactions of Smoking With rs833061 Polymorphism on the Risk of Non‐Alcoholic Fat Liver Disease in Hubei Han Population: A Preliminary Case‐Control Study,” Iranian Journal of Basic Medical Sciences 18, no. 11 (2015): 1112–1117. PMC4764113 26949499

[32] M. E. Rinella , J. V. Lazarus , V. Ratziu , et al., “A Multisociety Delphi Consensus Statement on New Fatty Liver Disease Nomenclature,” Journal of Hepatology 79, no. 6 (December 2023): 1542–1556, 10.1016/j.jhep.2023.06.003. 37364790

[33] N. Kapoor , R. Sahay , S. Kalra , et al., “Consensus on Medical Nutrition Therapy for Diabesity (CoMeND) in Adults: A South Asian Perspective,” Diabetes, Metabolic Syndrome and Obesity: Targets and Therapy 14 (April 2021): 1703–1728, 10.2147/DMSO.S278928. PMC8057793 33889005

[34] M. Assel , D. Sjoberg , A. Elders , et al., “Guidelines for Reporting of Statistics for Clinical Research in Urology,” Journal of Urology 201, no. 3 (March 2019): 595–604, 10.1097/JU.0000000000000001. PMC6600813 30633111

[35] National Center for Biotechnology Information (NCBI) , “dbSNP: rs3025039 Reference SNP Report. Build 157,” accessed October 26, 2026, https://www.ncbi.nlm.nih.gov/snp/rs3025039.

[36] B. D. Pardhe , S. Shakya , A. Bhetwal , et al., “Metabolic Syndrome and Biochemical Changes Among Non‐Alcoholic Fatty Liver Disease Patients Attending a Tertiary Care Hospital of Nepal,” BMC Gastroenterology 18, no. 1 (December 2018): 109, 10.1186/s12876-018-0843-6. PMC6035472 29980170

[37] M. F. K. Chowdhury , K. M. U. Zaman , M. A. Hasan , et al., “Pattern of Lipid Profile and Blood Pressure in Patients With Nonalcoholic Fatty Liver Disease (NAFLD),” Journal of Shaheed Suhrawardy Medical College 13, no. 1 (August 2022): 68–74, 10.3329/jssmc.v13i1.60935.

[38] J. Xing , X. Guan , Q. Zhang , S. Chen , S. Wu , and X. Sun , “Triglycerides Mediate Body Mass Index and Nonalcoholic Fatty Liver Disease: A Population‐Based Study,” Obesity Facts 14, no. 2 (2021): 190–196, 10.1159/000514848. PMC8138251 33780962

[39] C. H. Li , Y. T. Chou , W. C. Shen , et al., “Increased Risks of Different Grades of Non‐Alcoholic Fatty Liver Disease in Prediabetic Subjects With Impaired Fasting Glucose and Glucose Tolerance, Including the Isolated Glycosylated Hemoglobin Levels of 5.7%–6.4% in a Chinese Population,” Journal of Diabetes Investigation 11, no. 5 (September 2020): 1336–1343, 10.1111/jdi.13268. PMC7477498 32270583

[40] G. Hussein , A. A. Al Saud , A. A. Bashandi , et al., “Prevalence of Nonalcoholic Fatty Liver Disease at a Tertiary Care Center in Saudi Arabia,” Cureus 15, no. 10 (October 2023): 47896, 10.7759/cureus.47896. PMC10683321 38034241

[41] A. Oral , T. Sahin , F. Turker , and E. Kocak , “Relationship Between Serum Uric Acid Levels and Nonalcoholic Fatty Liver Disease in Non‐Obese Patients,” Medicina 55, no. 9 (September 2019): 600, 10.3390/medicina55090600. PMC6780528 31533345

