# Supplementary material for: Association of Vascular Endothelial Growth Factor A +936 C/T Polymorphism With Non‐Alcoholic Fatty Liver Disease in a Bangladeshi Population: A Case‐Control Study
Source: Health Sci Rep. 2026 Nov 5;9(11):e73494. doi: 10.1002/hsr2.73494 (PMC13735990; doi:10.1002/hsr2.73494)
Supplement: Supplementary file 1 [file HSR2-9-e73494-s001.docx]

**Supplementary Material**

**Manuscript Title: Association of vascular endothelial growth factor A +936 C/T polymorphism with non-alcoholic fatty liver disease in a Bangladeshi population: A case-control study**

**Supplementary Figure S1: Representative sequencing chromatograms showing CC, CT, and TT genotypes.**
